# Public perceptions and attitudes of drive-through pharmacy services: Insights from a cross-sectional survey in Saudi Arabia

**DOI:** 10.1097/MD.0000000000041118

**Published:** 2025-01-10

**Authors:** Safiya Salem Bakarman, Wajid Syed, Mohammad K Alharbi, Adel Bashatah, Mahmood Basil A. Al-Rawi

**Affiliations:** a Department of Community and Mental Health Nursing, College of Nursing, King Saud University, Riyadh, Saudi Arabia; b Department of Clinical Pharmacy, College of Pharmacy, King Saud University, Riyadh, Saudi Arabia; c Department of Nursing Administration and Education, College of Nursing, King Saud University, Riyadh, Saudi Arabia; d Optometry, College of Applied Medicals Science, King Saud University, Riyadh, Saudi Arabia.

**Keywords:** attitudes, drive-through services, opinions, perceptions, pharmacy services, Saudi Arabia

## Abstract

Disease-related fatalities in Saudi Arabia (SA) are on the rise, with 28% of all deaths attributed to cardiovascular diseases, followed by cancer, diabetes, and chronic respiratory disorders. In response to this issue, pharmacy dispensing practices have been significantly altered. One such innovative approach is the drive-through pharmacy service. This study aimed to assess the public perceptions and attitudes of drive-through pharmacy services living in Riyadh, SA. A cross-sectional, web-based study was conducted between October 2023 and December 2023 among adults living in Riyadh, SA to assess their perceptions and attitudes toward drive-through dispensing practice. A 19-item questionnaire, divided into 4 sections, was used to achieve the objectives. The first section focused on demographics and personal information. The second section included 3 questions about awareness, the population most benefited, and support. The final section assessed attitudes (5 items) of individuals toward drive-through pharmacy services. A response rate of 79.6% (n = 398) was achieved. A significantly high percentage of respondents, 86.2% (n = 343), reported being aware of drive-through pharmacy. Furthermore, 73.1% of them indicated that drive-through service benefits all populations, with 66.1% (n = 263) actually using the drive-through pharmacy service. In addition, 68.1% (n = 271) of individuals had positive perceptions. Positive perceptions were found to be higher among young adults aged 26 to 35 years compared with other age groups (*P* = .0001), among married individuals compared with unmarried individuals and others (*P* = .0001), as well as among housewives (*P* = .030). The results of the Student *t* test showed that males had a higher mean overall perception of drive-through pharmacy services (9.33) than females (9.26). Similarly, the analysis of variance analysis showed that individuals aged ≥46 years had a higher perception score (12.53) than other age groups. Furthermore, individuals with children had a lower perception (8.94) compared with those without children, indicating a statistically significant difference in mean perceptions of drive-through pharmacy based on gender (*P* = .002), age (*P* = .006), employment (*P* = .081), and presence of children (*P* = .001). Most respondents were aware of the drive-through pharmacy services and agreed that they would benefit the nation’s entire population. The majority of individuals also supported the expansion of drive-through pharmacy services.

## 1. Introduction

Saudi Arabia (SA) is experiencing a continuous increase in population and a high prevalence of chronic diseases which raises the need for constant medication management and increases dependency on pharmacies.^[1–5]^ To address this issue, pharmacy dispensing practices have changed significantly, and pharmacists are now integral to the delivery of health care services.^[6,7]^ To enhance pharmaceutical and preventative care services, SA has expanded the duties of pharmacists by allowing access to patient records from various hospitals and health care facilities through the Wasfaty program, using electronic dispensing.^[8,9]^ Despite the increasing workload, these practices require the introduction of new services for pharmacies, one of which is the drive-through service^[8,9]^ seen as a practical substitute for conventional counter service when it comes to administering drugs.^[7,10]^

Drive-through services, as defined by literature, are when a consumer obtains necessary items such as food, medication, or coffee through a window known as a drive-through window or a store that serves customers through a window, eliminating the need to exit their vehicle.^[11]^ During this process, cars line up in the drive-through lane for service.^[11]^ Drive-through services are not yet established in many countries, but well-developed countries like the United States, London, and South Africa have already implemented them.^[7,12,13]^ In SA, the first drive-through pharmacy was introduced in the Western region in April 2020 at the beginning of the Coronavirus disease 2019 pandemic. Since then, the number of pharmacies across the country offering drive-through services has grown rapidly.^[11]^ A recent report revealed that SA has introduced the world’s first drive-through automatic medicine dispenser, reducing the need for individuals and patients to visit hospitals and pharmacies, thus decreasing waiting times.^[14]^

Drive-through pharmacy services offer a wide variety of benefits for both pharmacists and their customers or patients.^[15–18]^ For patients, drive-through pharmacy services are considered fast and convenient compared with conventional methods. Waiting times are reduced, and patients receive rapid responses, improved dispensing, and availability of medicine.^[15–18]^ With busy schedules and hectic lifestyles, individuals are always looking for ways to save time. A drive-through allows customers to quickly pick up their medicine without having to park their cars, get out, and go into the pharmacy.^[15–18]^ Conversely, drive-through pharmacies enable pharmacists to confidently and precisely perform their duties.^[15–18]^ By using the drive-through window, pharmacists can quickly and reliably attend to the needs of their patients. Real-time support is available at the drugstore through a drive-through window.^[15–18]^

Despite the benefits of drive-through pharmacy services, literature also suggests some negative effects, such as the challenge of maintaining safe practices at the delivery level.^[15–18]^ In addition, the fast-paced nature of dispensing from the drive-through window may decrease interactions between patients and pharmacists, potentially leading to patients missing out on health education and counseling, which could negatively impact health outcomes.^[15–18]^ Patient and pharmacist communication is one of the most crucial components for enhancing satisfaction with care provided by pharmacists, patient compliance, and health outcomes. Patients are more likely to adhere to treatment plans and express greater satisfaction with the care they receive when they comprehend the nature of their condition and how it is being treated, as well as when they feel that the health care provider such as a pharmacist cares about their wellbeing. Despite the significance of patient and pharmacist communication in health care delivery at community pharmacies,^[19]^ it is often neglected. The implications of drive-through service may also be complicated when patients have new prescriptions.^[7,20]^ In addition, concern over drive-through pharmacists’ capacity to deliver the best possible patient care has grown.^[7,21]^ Patient counseling is also an important issue in reducing adverse drug events and improving adherence to the recommended regimen.^[7,21]^ Despite this pharmacist personnel issues also arise, as employees must accurately and rapidly process multiple orders simultaneously. Training staff members to operate a drive-through can be time-consuming and require significant effort.^[16]^

In this regard, public perceptions, opinions, and views regarding the drive-through dispensing practice are crucial for further enhancing the services or gaining insights from utilizing this service to improve its application as much as possible. Several reports from around the world have been published to explore the perceptions and acceptance of drive-through services among pharmacists and the general public. These reports have shown positive perceptions of drive-through pharmacy services, as well as support for the establishment of drive-through services at pharmacies. People believe that these drive-through services help to reduce the spread of infections.^[12,16,18]^ This finding can be utilized to design, enhance, and advance these services on a national and international level. In addition, the increasing workload of pharmacists requires the development of new services to improve pharmaceutical and preventative care. This study aimed to assess the public perceptions and attitudes of drive-through pharmacy services living in Riyadh, SA.

## 2. Methodology

### 2.1. Study design, sample, and data collection

A cross-sectional web-based study was conducted between October 2023 and December 2023 among adults living in Riyadh, SA, to assess perceptions, views, and opinions toward the drive-through dispensing practice. This study included adults aged ≥18 years, Saudi nationals currently living in the region, and willing to complete the survey by providing informed consent. Individuals who did not meet the inclusion criteria and those with incomplete responses were excluded from the study. Respondents were assured that the data would only be used for research purposes and would be kept confidential throughout the study. Before conducting the study, the protocols were reviewed and approved by the Ethics Committee for Human and Social Research at King Saud University, Riyadh, SA. Respondents who completed the questionnaires were considered to have given informed consent, and they had the right to withdraw from the study at any time. The study was conducted following the ethical standards established in the 1964 Declaration of Helsinki and its later amendments, or comparable ethical standards.

### 2.2. Sample size estimation

Similar to the previous studies,^[22–31]^ we calculated the required sample size using the Raosoft sample size calculator (http://www.raosoft.com/samplesize.html), considering the following assumptions: 95% confidence level, a margin of error of 5%, a population size of 7,820,551,^[22–31]^ and a 15% nonresponse rate. Therefore, the projected sample size was 385. However, we chose to survey 500 individuals, resulting in a sample size of 500 individuals.

## 3. Questionnaire design

The questionnaire used in this study was developed after reviewing available literature on perceptions, views, and opinions toward drive-through dispensing in pharmacies.^[10,12,32]^ It consisted of a 19-item questionnaire divided into 4 sections. The first section included 6 questions dedicated to demographics and personal information, such as gender, age, marital status, education, employment, and whether they have children. The second section comprised 3 questions about awareness of drive-through pharmacy presence, the population most benefited, and support toward drive-through pharmacy services. The third part of the study assessed individuals’ attitudes toward drive-through pharmacy services (5 items), such as trying drive-through pharmacy services, evaluating their experience, preferred method of collecting medicine, counseling at the drive-through pharmacy, and sources of information. The final section focused on perceptions of drive-through pharmacy services (5 items), including beliefs about efficiency, friendliness, and satisfaction. Perceptions-related questions were assessed on a 5-point Likert scale ranging from strongly agree to strongly disagree. A group of professionals, including researchers and professors from a pharmacy college, with experience in creating questionnaires, evaluated the initial draft. In addition, a pilot study was conducted among a randomly selected sample of 30 individuals to estimate the survey’s duration, readability of the questionnaires, and ease of administration. The pilot study’s outcome was excluded from the final analysis. According to the pilot study, the estimated time to complete the questionnaires was 16 minutes. The reliability of the questionnaire was checked using Cronbach α value, which revealed a value of 0.84 for perceptions items and 0.71 for attitudes and views, indicating strong relationships between the items on the scale. The data for this study were collected using convenience sampling, and an electronic tool was prepared and distributed using online platforms (WhatsApp, Twitter, and Facebook) to the targeted population. Participants were informed that their data would be used solely for research purposes, and confidentiality would be maintained throughout the study.

For 5-point Likert scale-based questions, a score of 5 was given to strongly agree, 4 for agree, 3 for neutral, 2 for disagree, and 1 for strongly disagree. The overall perception score for each question was computed, and the perception of drive-through was calculated by summing all perception questions. The total perception score was divided into two levels based on the median value. A positive perception score was considered when an individual scored ≥50, while <50 indicated a negative perception score.

### 3.1. Statistical analysis

Frequencies and percentages were calculated for each variable. To determine the association between categorical variables and demographics, a χ^2^/Fisher exact test was performed. To find out the differences in perceptions of drive-through pharmacy services and respondents’ characteristics *t* test and analysis of variance were applied. Logistic regression was used to assess how various sociodemographic characteristics were associated with respondents’ perceptions. The Statistical Package for Social Sciences, version 27.0 (SPSS Inc, Chicago, IL) was used for data analysis, with a *P* value of <.05 indicating statistical significance.

## 4. Results

Five hundred questionnaires were distributed to the general public living in Riyadh. A total of 448 individuals completed the questionnaires, but 50 were excluded due to not meeting the inclusion criteria. Finally, 398 responses were included in the final analysis, resulting in a response rate of 79.6%. The profile of the study sample is shown in Table 1. The sample consisted of 68.6% male and 31.4% female participants, with 24.4% falling in the age range of 31 to 45 years. In addition, 53.5% were married, 70.1% had at least a university-level qualification, and 52.5% reported being employed. The majority of respondents (78.1%) had children.

**Table 1:** Sociodemographic characteristics of the study respondents (n = 398).

Characteristics	Frequency, n (%)
Gender	
Male	273 (68.6)
Female	125 (31.4)
Age (in yr)	
20–24	131 (32.9)
25–30	71 (17.8)
31–35	97 (24.4)
36–40	55 (13.8)
41–45	31 (7.8)
>46	13 (3.3)
Marital status	
Single	167 (42.0)
Married	21 3(53.5)
Divorced	09 (2.3)
Widowed	09 (2.3)
Educational degree	
Primary	55 (13.8)
Secondary	64 (16.1)
University	279 (70.1)
Employment	
Housewife	21 (5.3)
Unemployed	52 (13.1)
Employed	209 (52.5)
Student	116 (29.2)
Having children	
Yes	311 (78.1)
No	87 (21.9)

### 4.1. Awareness of drive-through pharmacy services

**Figure 1.**
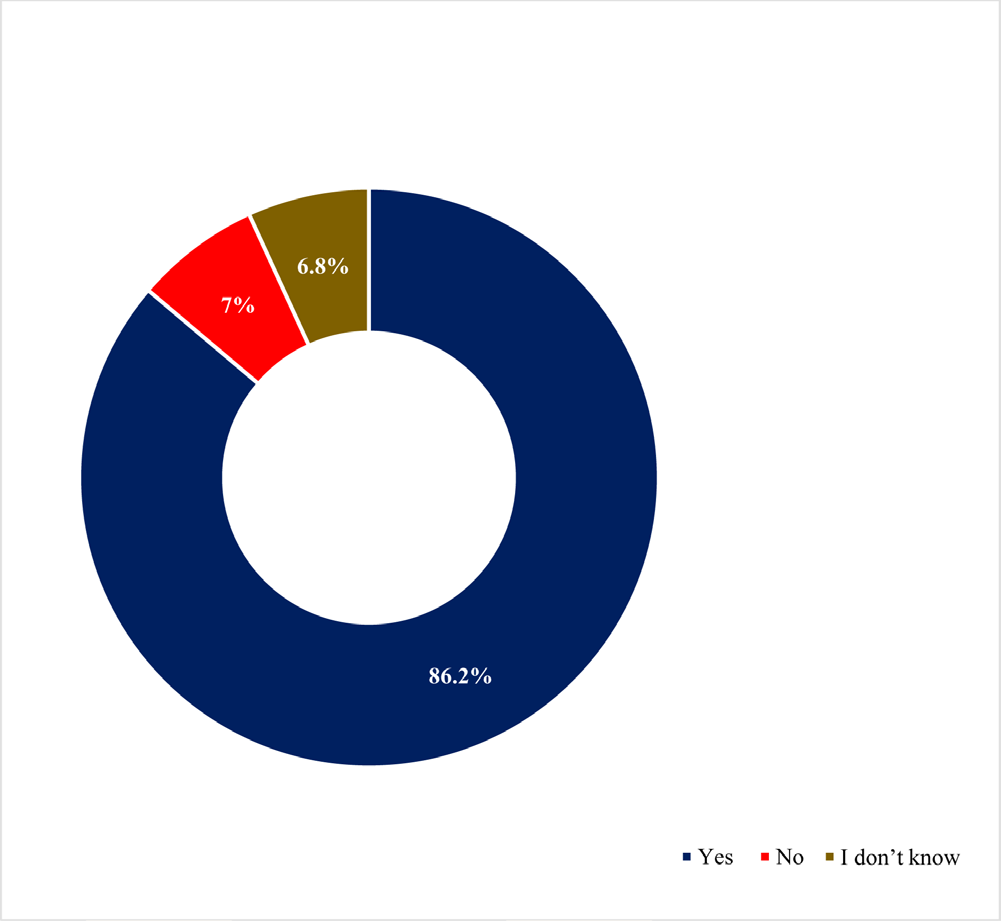
Have you seen the presence of drive-through pharmacy services.

**Figure 2.**
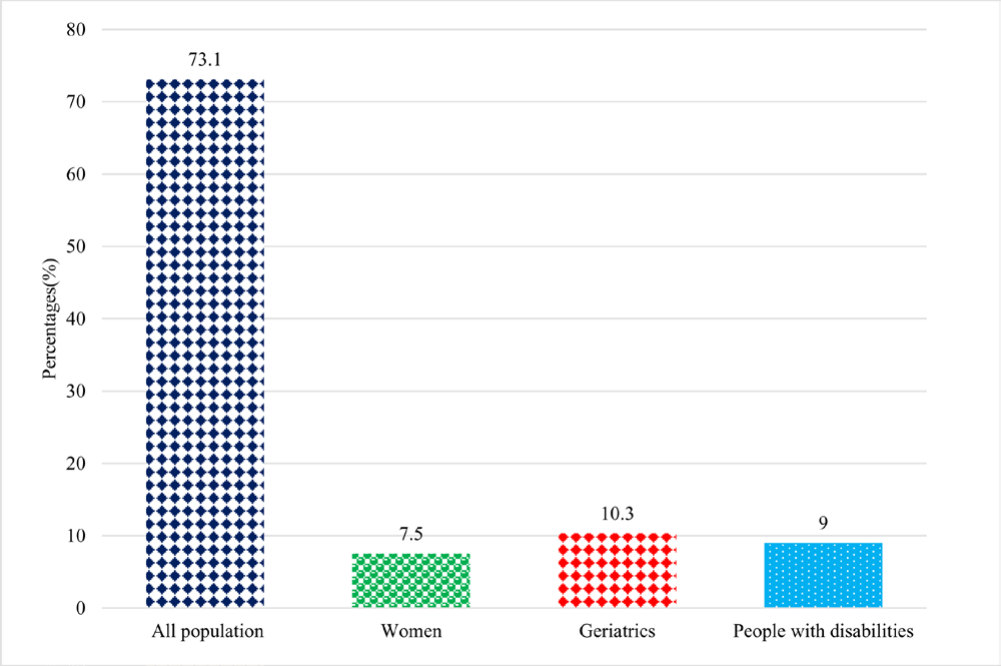
Categories of the population benefit from drive-through pharmacy services.

**Figure 3.**
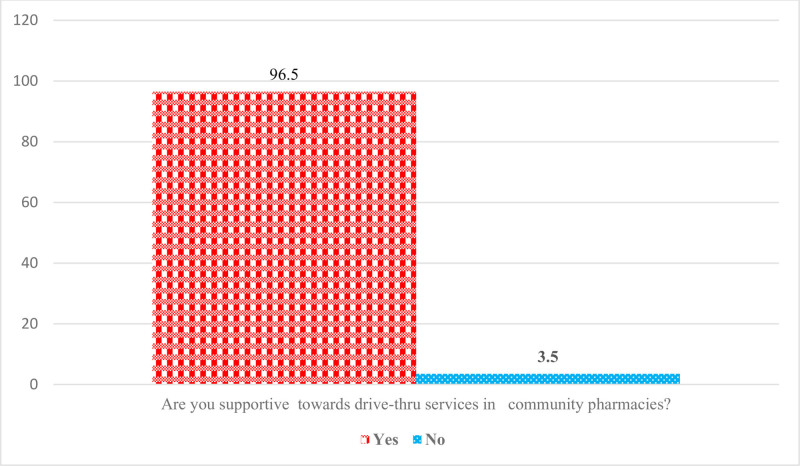
Respondents’ views toward drive-through pharmacy.

A significantly high proportion of respondents reported being aware of drive-through pharmacy services, with 86.2% (343) acknowledging their existence (Fig. 1). Among them, 73.1% believed that these services benefit all populations, while 10.3% stated they are beneficial for the geriatric population, and 9% said people with disabilities benefit from drive-through pharmacy services (Fig. 2). Furthermore, the majority expressed positive views and indicated their support for drive-through services throughout the SA (Fig. 3). In terms of attitudes, 66.1% of the respondents said they had previously used the drive-through pharmacy service, 34.7% stated they would use the drive-through window to request a medication order; additionally, 48.2% of them said the drive-through window was their preferred method of obtaining information about the medications or counseling they were using. The detailed responses of attitudes toward drive-through pharmacy are given in Table 2.

**Table 2:** Respondents’ attitudes of drive-through pharmacy (n = 398).

Variables	Frequency, n (%)
Have you tried at least 1 drive-through pharmacy service?	
Yes	263 (66.1)
No	102 (25.6)
Not applicable	33 (8.3)
If yes, how was your experience with the drive-through pharmacy service	
Excellent	172 (65.4)
Good	69 (26.2)
Fair	19 (7.2)
Poor	03 (1.1)
If you are going to request an order at a pharmacy using a drive-through service, what is your preferred method to do that order?	
Drive-through window	138 (34.7)
WhatsApp	59 (14.8)
Over the phone	165 (41.5)
Online through application	30 (7.5)
Using an email	06 (1.5)
If you are going to use a drive-through service at a pharmacy, what is your preferred method to get information about your medications (counseling; >1 answer is possible)	
Briefly through the drive-through window	192 (48.2)
Printed brochure given with the order	115 (28.8)
Written on WhatsApp	61 (15.3)
Verbally over the phone	198 (49.7)
Through a personal visit	143 (35.9)
Through email	17 (4.27)
Where did you get information regarding the drive-through pharmacy? (>1 answer is possible)	
Pharmacy staff	132 (33.1)
Doctors	83 (20.8)
Leaflets	102 (25.6)
Television	168 (42.2)
Internet	251 (63)
Friends or Colleagues	250 (62.8)
Do not know	21 (5.2)

### 4.2. Factors associated with the experience of using drive-through pharmacy services (n = 263)

The associations between the experience of using drive-through pharmacy services and factors such as age, gender, employment, education, and marital status, were determined using χ^2^ and Fisher exact tests at the <0.05 level (Table 3). Results showed that age (*P = .*0001), and marital status (*P = *.05), had a significant association with the experience of using drive-through pharmacy services suggesting that age and marital status are significant factors affecting the experience of using drive-through pharmacy services as shown in Table 3.

**Table 3:** Association between participants, and demographic characters associated with the experience of using drive-through pharmacy services (n = 263).

Variables	Excellent, n (%)	Good, n (%)	Fair, n (%)	Poor, n (%)	χ^2^	*P* value
Gender					2.688	.442
Male	118 (68.6)	45 (65.2)	15 (78.9)	03 (100.0)		
Female	54 (31.4)	24 (34.8)	04 (21.1)	0 (0.0)		
Age						.0001
18–25	66 (38.4)	27 (39.1)	5 (26.3)	0 (0.0)	38.884	
26–30	27 (15.7)	16 (23.2)	4 (21.1)	1 (33.3)		
31–35	41 (23.8)	9 (13.0)	2 (10.5)	2 (66.7)		
36–40	20 (11.6)	8 (11.6)	2 (10.5)	0 (0.0)		
41–45	15 (8.7)	9 (13.0)	2 (10.5)	0 (0.0)		
>46	3 (1.7)	0 (0.0)	4 (21.1)	0 (0.0)		
Marital status						.050
Divorced	2 (1.2)	0 (0.0)	2 (10.5)	0 (0.0)	16.791	
Married	88 (51.2)	35 (50.7)	12 (63.2)	3 (100.0)		
Single	78 (45.3)	33 (47.8)	5 (26.3)	0 (0.0)		
Widowed	4 (2.3)	1 (1.4)	0 (0.0)	0 (0.0)		
Education					8.238	.221
Primary	10 (5.8)	7 (10.1)	2 (10.5)	1 (33.3)		
Secondary	29 (16.9)	15 (21.7)	6 (31.6)	0 (0.0)		
University	133 (77.3)	47 (68.1)	11 (57.9)	2 (66.7)		

χ^2^ = Pearson χ^2^.

### 4.3. Perceptions of drive-through pharmacy services

In this study, the majority (83.5%) of the respondents believed that drive-through pharmacy services are a friendly service offered by pharmacies. In addition, 78.4% agreed or strongly agreed that the introduction of drive-through services makes pharmacy services more efficient. Furthermore, 74.9% of respondents stated that drive-through pharmacy services would increase satisfaction with the pharmacy field. Moreover, 89.2% of respondents expressed support for the introduction of drive-through services to pharmacies. It is worth noting that the vast majority strongly agreed that they would welcome the establishment of drive-through pharmacy services in South Australia. The detailed responses of the respondents regarding their perceptions toward drive-through pharmacy services are provided in Table 4.

**Table 4:** Perceptions of drive-through pharmacy services.

Variables	Strongly disagree/disagree, n (%)	Neutral, n (%)	Strongly agree/agree, n (%)	Mean (SD), 95% CI
I believe the introduction of drive-through services makes pharmacy services more effective	29 (7.3)	57 (14.3)	312 (78.4)	3.14 ± 1.34 Lower bound 3.00 Upper bound 3.27
I believe that drive-through pharmacy service is a friendly service provided by the pharmacies	32 (8.0)	34 (8.5)	332 (83.5)	2.78 ± 1.36 Lower bound 2.64 Upper bound 2.91
I believe that drive-through pharmacy service may improve satisfaction with the pharmacy profession, by reducing waiting time	24 (6.0)	76 (19.1)	298 (74.9)	2.73 ± 1.25 Lower bound 2.60 Upper bound 2.85
I am supportive of the introduction of drive-through service to pharmacy practice	09 (2.3)	34 (8.5)	355 (89.2)	3.49 ± 1.27 Lower bound 3.36 Upper bound 3.62
I am supportive of creating pharmacies with drive-through services all over SA	18 (4.6)	48 (12.1)	332 (83.4)	3.59 ± 1.30 Lower bound 3.46 Upper bound 3.72

CI = confidence interval, SA = Saudi Arabia, SD = standard deviation.

**Figure 4.**
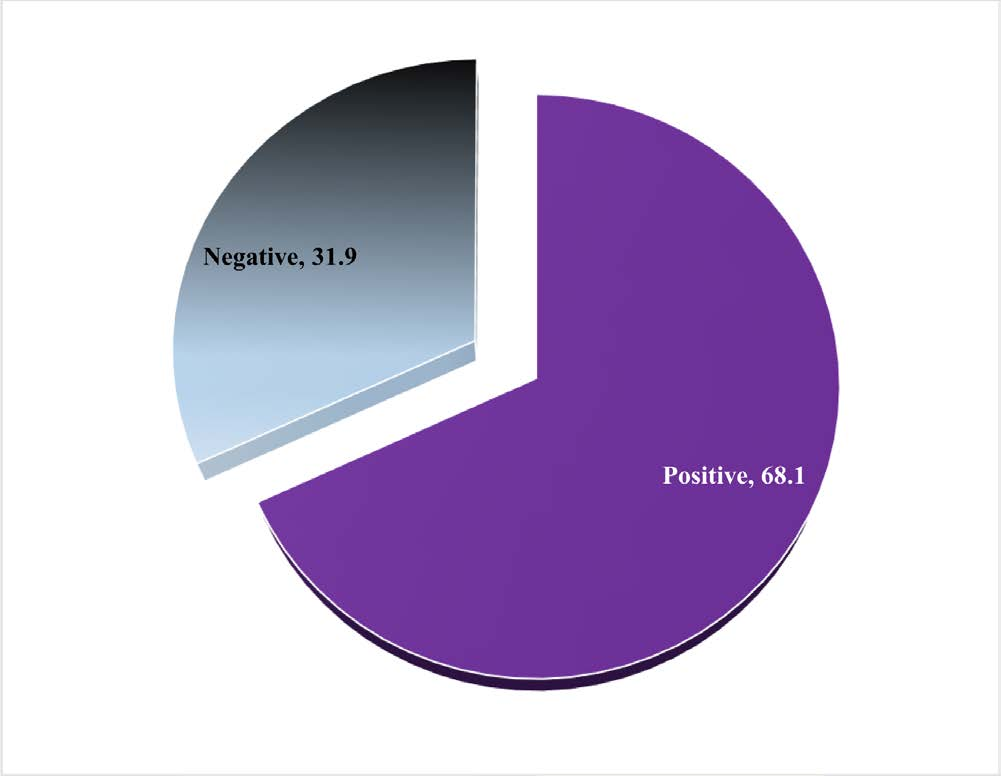
Levels of perceptions of drive-through pharmacy services.

In this study, 68.1% (n = 271) of individuals were found to have positive perceptions, while 31.9% of them reported negative perceptions toward drive-through pharmacy services (Fig. 4).

To demonstrate the relationship between demographic characteristics such as gender, age, marital status, and the perception levels of drive-through pharmacy services, a χ^2^ or Fisher exact test was conducted. The data indicate that perceptions of drive-through pharmacy were significantly linked to age. Positive perceptions were more common among young adults aged 26 to 35 years compared with other age groups (*P* = .0001). Similarly, positive perceptions were higher among married individuals compared with unmarried and others (*P* = .0001), as well as among housewives (*P* = .030), suggesting that marital status and professional classification were significantly associated with perceptions, as outlined in Table 5.

**Table 5:** Association between participants’ demographic characteristics and perception levels of drive-through pharmacy services.

Variables	Negative, n (%)	Positive, n (%)	χ^2^	*P* value
Gender			1.282	.258
Male	92 (72.4)	181 (66.8)		
Female	35 (27.6)	90 (33.2)		
Age			21.747	.0001*
>46	1 (0.8)	12 (4.4)		
18–25	60 (47.2)	71 (26.2)		
26–30	15 (11.8)	56 (20.7)		
31–35	23 (18.1)	74 (27.3)		
36–40	19 (15.0)	36 (13.3)		
41–45	9 (7.1)	22 (8.1)		
Education				
Primary	11 (8.7)	44 (16.2)	6.983	.030
Secondary	16 (12.6)	48 (17.7)		
University	100 (78.7)	179 (66.1)		
Employment				.0001
Housewife	49 (38.6)	160 (59.0)	18.692	
Unemployed	20 (15.7)	32 (11.8)		
Employed	53 (41.7)	63 (23.2)		
Student	5 (3.9)	16 (5.9)		
Marital status				
Single	71 (55.9)	96 (35.4)	17.301	.0001*
Married	54 (42.5)	159 (58.7)		
Divorced	02 (1.6)	07 (2.6)		
Widowed	0 (0.0)	09 (3.3)		

χ^2^ = Pearson χ^2^.

* Fisher exact test.

### 4.4. Differences in perceptions of drive-through pharmacy services among respondents and their characteristics

To determine variances in perceptions of drive-through pharmacy based on respondents’ age, gender, marital status, education, employment, and parental status, a Student *t* test (for categorical variables with <2 categories) and analysis of variance (for >2 categorical variables) were used. The analysis indicated that males had a higher mean perception of drive-through pharmacy services (9.33) than females (9.26) (*P* = .002). Similarly, individuals aged ≥46 years had a higher perception score (12.53) than other age groups, showing a significant difference (*P* = .006). In addition, unemployed individuals had a higher mean perception of drive-through pharmacy services (9.78) than employed individuals and others (*P* = .081). Conversely, individuals with children had a lower average perception score (8.94) compared with those without children, indicating a statistically significant difference (*P* = .001) as presented in Table 6.

**Table 6:** Differences in perceptions of drive-through pharmacy services among respondents and their characteristics.

Variables	Count (n)	Mean (SD)	SEM	SE	*F*	*t*	*P* value
Gender						0.171	.002*
Male	273	9.33 (4.21)	0.25				
Female	125	9.26 (2.50)	0.22				
Age							.006
18–25	131	8.83 (4.28)		0.37	3.337		
26–30	71	8.95 (2.14)		0.25			
31–35	97	9.84 (4.18)		0.42			
36–40	55	9.65 (3.65)		0.49			
41–50	31	8.48 (2.17)		0.39			
>46	13	12.53 (3.15)		0.87			
Marital status							
Single	167	9.38 (4.47)		0.34	0.197		.898
Married	213	9.21 (3.21)		0.22			
Divorced	09	9.66 (3.04)		1.01			
Widowed	09	10.0 (1.00)		0.33			
Education level							
Primary	55	9.12 (2.30)		0.31	.161		.851
Secondary	64	9.51 (3.12)		0.39			
University	279	9.30 (4.11)		0.24			
Employment							
Housewife	209	9.63 (3.60)		0.24	2.264		.081
Unemployed	52	9.78 (4.10)		0.56			
Employed	116	8.62 (4.00)		0.37			
Student	21	8.76 (2.27)		0.49			
Having a child							
Yes	311	8.94 (3.34)	0.18			−3.702	.001*
No	87	10.60 (4.76)	0.51				

SD = standard deviation, SE ***=*** standard error, SEM ***=*** standard error mean.

* *t* test.

## 5. Discussion

According to the current findings, a significantly higher percentage of participants (86.2%) reported being familiar with drive-through pharmacy services, and 73.1% of them felt that these services were beneficial to all age groups. In addition, 68.1% of participants expressed positive perceptions, and 83.5% of them believed that the pharmacies provide friendly service through drive-through pharmacy services. Finally, 78.4% of participants agreed that the introduction of drive-through services has increased the efficiency of pharmacy services. Moreover, 65.4% of the people who utilized the drive-through pharmacy service reported that it was an excellent experience, making up 66.1% of the total. In addition, the majority expressed favorable attitudes and perceptions and said that they supported drive-through services throughout the SA. The current findings revealed a relatively high awareness of drive-through pharmacies compared with earlier studies.^[10,12,32]^ For instance, a Malaysian study reported that half of the respondents were unaware of the presence of drive-through services.^[32]^ In Jordan, another recent study revealed limited awareness of drive-through pharmacy services.^[10]^ Similarly, another study in 2020 among Saudi adults revealed very low levels of awareness.^[33]^ Another study conducted in Malaysia in 2015 found that approximately 60% of respondents were aware of the presence of a drive-through pharmacy service and recognized its importance for public use.^[34]^ Despite undergoing significant development through Vision 2030, SA is also experiencing rapid advancements in health care.^[35]^ The Coronavirus disease 2019 pandemic has heightened awareness about the importance of protective measures, such as avoiding large crowds.^[36,37]^ As a result, people in SA and other countries are increasingly turning to online services and contactless methods to make purchases.^[7,12,32,38]^ These factors are likely contributing to the growing popularity of drive-through services. The high level of awareness observed in this study may be attributed to cultural differences, improvements in health care, and the effectiveness of the questionnaires used.

In this study, the majority of the respondents reported that drive-through pharmacy services benefit the population. However, 10.3% of the respondents believed that it is particularly beneficial for geriatric patients. Similar findings were reported in other studies.^[12,32,33]^ For example, a study conducted in 2020 found that 78% of respondents agreed that drive-through pharmacy services are beneficial for the population, especially during pandemics. The same study also highlighted that drive-through pharmacy services are particularly beneficial for people with disabilities.^[33]^ Similarly, 75.8% of the Malaysian population agreed that drive-through pharmacy services are beneficial for all populations, while 13.8% reported it as more suitable for individuals with disabilities.^[32]^ Furthermore, the current findings contradict earlier research, where the author claimed that drive-through pharmacy services were mainly beneficial to specific segments of the population, such as the elderly, disabled, and ill patients.^[32,39]^ In Malaysia, individuals who used the drive-through pharmacy service were satisfied with the service provided by pharmacists.^[20]^ In SA, literature published in 2020 suggested a crucial need to support pharmacies with drive-through pharmacy services.^[33]^

In terms of usage, 65.3% of participants said they have used drive-through pharmacy services. This contradicts an earlier study that found that only 5.5% of Malaysians who utilized drive-through pharmacy services said they had a positive experience, while only 9.0% of them did so.^[32]^ However, in the current study, utilization of drive-through services is relatively high. This may be because the majority of respondents were young and educated, which may increase the likelihood of increased awareness and utilization of drive-through pharmacy services. In addition, SA is a developed nation that has adopted various advancements in health care. The differences in findings between the current and previous studies could be attributed to the presence of multiple drive-through pharmacy services and their awareness.

In addition to the high awareness and utilization of drive-through pharmacy services, 52% of the respondents had positive perceptions, while 48% (n = 191) reported negative perceptions. These results were consistent with previous studies.^[12,32]^ For example, in Malaysia, the majority of respondents had a positive perception of drive-through pharmacy services.^[32]^ Similarly, a systematic review of 55 studies found favorable perceptions and attitudes toward drive-through and extended pharmacy services.^[32]^ Previous research in Jordan also showed favorable customer awareness and perceptions of drive-through pharmacy services.^[12]^ Attitudes and perceptions, influenced by an individual’s thoughts, play a significant role in decision-making and readiness to engage in a particular behavior.^[40]^ They assess a service based on cognitive thoughts, beliefs, values, and emotions toward the service.^[41]^ Attitudes and perceptions play a significant role in the decision-making process when deciding to use a service or purchase a product.^[41]^

In this study, 83% of the respondents perceived drive-through pharmacy services as a friendly offering from pharmacies, while 78.4% believed that the introduction of drive-through services made pharmacy services more efficient. In addition, 74.9% stated that drive-through pharmacy services increased customer satisfaction by reducing waiting times. These findings align with earlier research indicating that drive-through services are viewed positively and enhance satisfaction with the pharmacy industry.^[12,32]^ Participants also agreed that pharmacists can balance the business aspect of their jobs with patient health.^[32]^ In Jordan, a majority supported the introduction of drive-through services to pharmacy practice and viewed it as a friendly service.^[12]^ This positive perception is likely due to the convenience and speed of obtaining medications without long wait times.

In this study, the perception of drive-through pharmacies was found to be significantly associated with respondents’ age, marital status, education, and employment. Age and marital status were significantly associated with the experience of using drive-through pharmacy services. Currently, there are no studies examining the differences in perception scores between individual traits and drive-through pharmacies. Some international studies have evaluated drive-through pharmacy services among the public^[32,33]^ and pharmacists^[12]^ For example, the public literature concluded that perceptions were associated with age, nationality, and working in the health sector. From the pharmacist perspective, Abu Farha et al revealed that pharmacists working in chain pharmacies had a better perception of drive-through pharmacy service compared with pharmacists working in independent pharmacies. In the Saudi context, there is a study that assessed drive-through pharmacy perceptions among pharmacists^[2]^ and found that negative perception was moderately negatively correlated with positive perception and weakly negatively correlated with satisfaction with drive-through services. In addition, previous studies have also shown that a positive perception is positively associated with satisfaction with drive-through services.^[2]^ The fact that seniors, who are older and married, may use more pharmacy services compared with others may have contributed to the significant difference in perception scores in this study.

This study has several limitations, First, the data were specific to one region of SA and only accessible to citizens, potentially limiting its relevance to other regions particularly rural areas. In addition, the cross-sectional design of the study prevents the inference of causality. Second, the use of self-reported questionnaires may have introduced recall or measurement biases, suggesting the need for alternative data collection methods such as interviews or focus groups in future studies. Third, the use of online platforms may have also excluded certain populations, such as the elderly or those without internet access. Convenience sampling was another limitation of the study’s sample. It is recommended that future studies be conducted with a larger sample size including all regions in SA to strengthen the evidence. In addition, a more focused discussion on refining the sampling technique (eg, stratified random sampling) would be more helpful. Despite these limitations, the research highlights the importance of increasing awareness and satisfaction with drive-through pharmacy services, emphasizing the need for pharmacists to improve their knowledge of customer service and the implications of their skills. This study provides a solid foundation for further research in this area.

## 6. Conclusion

A high proportion of respondents were aware of drive-through pharmacy services, had positive perceptions, and agreed that they would benefit the overall population. The majority of individuals also support the extension of drive-through pharmacies across the SA. Furthermore, 66.1% of them reported using at least 1 drive-through pharmacy. Factors such as age, marital status, and employment status of the respondents were significantly associated with perceptions of the drive-through pharmacy. In addition, age and marital status had a significant association with the experience of using drive-through pharmacy services. Although using this service can be associated with poor communication between pharmacists and patients, leading to pharmacists being unable to counsel patients properly, it is crucial to address these concerns and implement structural changes to the pharmacy system. To address this issue, standardization of pharmacies and separate prescription pickup windows are advised. Finally, recognizing the fundamental challenges of providing these services and improving pharmacists’ competence through extra training programs allows them to provide such services more efficiently. Further evaluations of this practice are advised in the future to address these difficulties and offer standardized suggestions for good pharmacy service delivery among stakeholders and organizations. More research on the implementation of drive-through pharmacy services is needed to better assist decision-makers in determining if they would contribute to pharmaceutical therapy without requiring a large budget.

## Acknowledgments

The authors of this study extend their appreciation to the Researchers Supporting Project (Project number RSP2024R378), King Saud University, Riyadh, Saudi Arabia.

## Author contributions

**Data curation:** Safiya Salem Bakarman, Mohammad K Alharbi, Mahmood Basil A. Al-Rawi.

**Formal analysis:** Safiya Salem Bakarman, Wajid Syed, Mohammad K Alharbi, Adel Bashatah, Mahmood Basil A. Al-Rawi.

**Funding acquisition:** Safiya Salem Bakarman, Wajid Syed, Mohammad K Alharbi, Adel Bashatah, Mahmood Basil A. Al-Rawi.

**Investigation:** Safiya Salem Bakarman, Wajid Syed, Mohammad K Alharbi, Adel Bashatah, Mahmood Basil A. Al-Rawi.

**Writing—original draft:** Safiya Salem Bakarman, Wajid Syed, Adel Bashatah.

**Writing—review & editing:** Safiya Salem Bakarman, Wajid Syed, Mohammad K Alharbi, Adel Bashatah.

**Supervision:** Wajid Syed, Mahmood Basil A. Al-Rawi.

**Validation:** Wajid Syed, Mahmood Basil A. Al-Rawi.

**Visualization:** Wajid Syed, Adel Bashatah.

**Methodology:** Mohammad K Alharbi, Mahmood Basil A. Al-Rawi.

**Conceptualization:** Adel Bashatah, Mahmood Basil A. Al-Rawi.

**Resources:** Adel Bashatah, Mahmood Basil A. Al-Rawi.

**Project administration:** Mahmood Basil A. Al-Rawi.

**Software:** Mahmood Basil A. Al-Rawi.
